# Exploring the impacts of low‐cost digital technologies on cognitive impairment

**DOI:** 10.1002/alz70860_098766

**Published:** 2025-12-23

**Authors:** Arman Hajikarim‐Hamedani, Setareh Rassa, Anahita Tarki, Maryam Noroozian

**Affiliations:** ^1^ Cognitive Neurology, Dementia and Neuropsychiatry Research Center, Tehran University of Medical Sciences, Tehran, Tehran, Iran (Islamic Republic of); ^2^ Cognitive Neurology, Dementia and Neuropsychiatry Research Center (CNNRC), Tehran, Tehran, Iran (Islamic Republic of)

## Abstract

**Background:**

Currently, more than 55 million people have dementia worldwide, and by 2050, an estimated 135 million people will suffer from dementia. On the other hand, In recent years, life has digitalized daily, with high levels of technical revolution and global publication of information and communication technology (ICT). Digital care services, ICT applications and mobile devices provide a plenty of benefits to reduce cognitive dysfunctions, However, accessing technology is not equal in different countries since many developing countries have lots of obstacles and limitations in using the internet, online resources, social media, and high‐cost assistive technologies; therefore, elderlies in low and middle‐income countries such as Iran, cannot access the benefits that digital technologies are offered, also due to the globally digitalization of researches besides lower digital familiarity among older adults in these countries, the knowledge around this area is remained unclear. This paper aims to evaluate and highlight the impacts of affordable and available technologies, that can be accessed in developing countries, on cognitive impairment.

**Method:**

Following PRISMA guidelines, studies from PubMed, Scopus, Web of Science, and PsycInfo (1991‐2025) were reviewed. Data were extracted using EndNote 21, focusing on demographics, study characteristics, population details, behavioral symptoms, and cognitive symptoms. Methodological quality was assessed using modified Cochrane and Effective Public Health Practice Project tools.

**Result:**

We found using low‐cost technology and digital tools can enhance the quality of life in cognitive impairment, for instance health and social care online services, social media which causing connection and reducing loneliness, GPS tracking that helps with wayfinding and safe walking, applications that designed to improve memory, assessing cognitive impairment in early‐stages, helping caregivers access information online, setting reminders for medications and other therapies, overall these technologies will higher the confidence and independence in daily living activities of patients with cognitive impairment.

**Conclusion:**

Using digital technologies and medical apps for a relatively low cost showed multiple beneficial effects in preserving cognition in older adults and patients with dementia from low‐ to mid‐income countries.

